# Vaccination with Altered Peptide Ligands of a *Plasmodium berghei* Circumsporozoite Protein CD8 T-Cell Epitope: A Model to Generate T Cells Resistant to Immune Interference by Polymorphic Epitopes

**DOI:** 10.3389/fimmu.2017.00115

**Published:** 2017-02-14

**Authors:** Gabriela Minigo, Katie L. Flanagan, Robyn M. Slattery, Magdalena Plebanski

**Affiliations:** ^1^Vaccine and Infectious Diseases Laboratory, Department of Immunology and Pathology, Monash University, Melbourne, VIC, Australia; ^2^Global and Tropical Health Division, Menzies School of Health Research, Charles Darwin University, Darwin, NT, Australia; ^3^School of Medicine, University of Tasmania, Hobart, TAS, Australia; ^4^Diabetes Laboratory, Department of Immunology and Pathology, Monash University, Melbourne, VIC, Australia

**Keywords:** cross-reactivity, altered peptide ligand, antagonism, T cell, dendritic cell, vaccine, *Plasmodium*, malaria

## Abstract

Many pathogens, including the malaria parasite *Plasmodium falciparum*, display high levels of polymorphism within T-cell epitope regions of proteins associated with protective immunity. The T-cell epitope variants are often non-cross-reactive. Herein, we show in a murine model, which modifies a protective CD8 T-cell epitope from the circumsporozoite protein (CS) of *Plasmodium berghei* (SYIPSAEKI), that simultaneous or sequential co-stimulation with two of its putative similarly non-cross-reactive altered peptide ligand (APL) epitopes (SYIPSAEDI or SYIPSAEAI) has radically different effects on immunity. Hence, co-immunization or sequential stimulation *in vivo* of SYIPSAEKI with its APL antagonist SYIPSAEDI decreases immunity to both epitopes. By contrast, co-immunization with SYIPSAEAI has no apparent initial effect, but it renders the immune response to SYIPSAEKI resistant to being turned off by subsequent immunization with SYIPSAEDI. These results suggest a novel strategy for vaccines that target polymorphic epitopes potentially capable of mutual immune interference in the field, by initiating an immune response by co-immunization with the desired index epitope, together with a carefully selected “potentiator” APL peptide.

## Introduction

Foreign epitopes complexed with host MHC molecules are the target of recognition by cognate antigen-specific T cells. The development of effective preventive vaccines requires the induction of long-lasting immunity in the human population, with the capacity to induce responses to naturally occurring strains of a pathogen bearing different variants of protective epitopes. Pathogens bearing variant epitopes can evade or skew the immune system in a variety of ways leading to loss of protective immunity. A direct form of immune evasion involves the mutation of amino acid residues that are required for peptide binding to MHC. Other more sophisticated forms of polymorphic immune evasion also exist [reviewed in Ref. (1)]. Altered peptide ligand (APL) antagonism involves the concurrent presentation of selected closely related epitope variants. This can inhibit T cell effector functions such as cytokine production, cytotoxicity, or proliferation. The use of APL ligands for immune evasion has been well documented across human immunodeficiency virus (HIV), hepatitis B and C (HBV and HBC), and malaria parasite infections [reviewed in Ref. (1)]. Another form of immune evasion, known as “immune interference,” also involves the concurrent or sequential presentation of related epitope variants to T cells. Immune interference results a failure to induce memory T cells from naive precursors. CD8 T-cell epitope variants causing APL antagonism and immune interference have been identified in *Plasmodium falciparum* and are major contributors to the parasite population structure observed in malaria-endemic regions of the world (2, 3).

Variant-specific immunity has been documented for the lead preerythrocytic malaria vaccine, RTS,S, which contains a single polymorphic variant of the circumsporozoite protein (CS) of *P. falciparum* (4). However, vaccines that target individual variants provide limited or short-lasting benefit in protection from malaria. This occurs because the non-targeted parasite variants take over the vacated niches in the vaccine-treated host population [discussed in Ref. (1)]. Attempts to provide more broadly cross-reactive responses against malaria have included mixing polymorphic variants of a single antigenic protein into one vaccine formulation, for example, including both the 3D7 and FC27 variants of merozoite surface protein 2 from *P. falciparum* into a single recombinant erythrocytic-stage vaccine (5); or mixing multiple target proteins into one formulation, for example, including the two key preerythrocytic-stage antigens CS and thrombospondin-related adhesive protein (TRAP) (6). Both attempts yielded disappointing results, with the combination of antigens showing little increase, and in the case of CS and TRAP, even a decrease, in immunity. As an alternative, recent studies have evaluated whether changing the adjuvant used to deliver a protective preerythrocytic *Plasmodium berghei* CD8 T-cell epitope (SYIPSAEKI) in murine models could broaden the pattern of T cell cross-reactivity (7). SYIPSAEKI (KI) is the immunodominant CD8 T-cell epitope of the *P. berghei* circumsporozoite (CS) protein, and the presence of vaccine-induced IFNγ-producing splenic T cells to this epitope correlates with protective efficacy (8). The contact T cell receptor (TCR) amino acid residue has been identified to be position 8, and in recent studies engineered amino acid changes to this position resulted in the generation of useful variants to model how an amino acid change can lead to loss of T cell cross-reactivity, without a decrease in MHC-binding capacity (8). Disappointingly, although both pro-inflammatory (montanide and poly I:C) and non-inflammatory nanoparticle-based vaccines induced comparable and robust peptide-specific responses to KI, they induced limited cross-reactivity to variants SYISAEDI (DI) or SYIPSAEAI (AI) (8). The limited cross-reactivity was also shown not to be due to holes in the naïve T cell repertoire, since vaccines formulated with each APL individually (including KI, AI, or DI) were capable of inducing robust immune responses to the immunizing index APL (7). While neither DI nor AI are natural variants, inclusion of such APLs in malaria vaccines has the potential to induce a broader spectrum of cross-reactive responses different from immunization with the index epitope alone. Conversely, if any of the pooled variants had antagonistic APL properties, such an approach could restrict the spectrum of responses, or turn off preexisting immunity, thus inducing immune interference, as has been described for naturally occurring variants of CS T-cell epitopes (9). Testing the consequences of immunizing with combinations or mixtures of APL variants has not been explored in such previous studies.

Understanding the mechanism by which immune interference promotes parasite survival and influences parasite population dynamics is central to successful vaccine development. In the present study, a murine model was utilized to dissect the complex patterns of immunity induced by well characterized selected APL variants of the *P. berghei* CD8 T-cell epitope KI, DI, and AI. The findings demonstrate a novel vaccination strategy that broadens T cell immunity and is resistant to being “turned off” by immune interference. This new vaccination strategy is likely to be of utility in tackling diverse pathogens beyond malaria that utilizes APLs for immune evasion, such as HIV and hepatitis C [reviewed in Ref. (1)].

## Animals and Methods

### Animals

Female BALB/c (H-2^d^) mice, 6–10 weeks of age were sourced from the Walter and Eliza Hall Institute, Melbourne, VIC, Australia, or bred at the Austin Research Institute Biomedical Animal Research Laboratory. The study was approved by Austin Research Institute Animal Care and Use Committee approved all animal procedures.

### Peptides

The immunodominant CD8 epitope of *P. berghei* circumsporozoite protein SYIPSAE**K**I, its variants SYIPSAE**D**I and SYIPSAE**A**I, and the influenza CD8 epitope NPKd (TYQRTRALV) were synthesized by to >99% purity by Auspep (Melbourne, VIC, Australia). Peptides were confirmed to be non-toxic at the doses used by incubation of splenocytes overnight and assessment of impairment of ConA responses by ELISPOT.

### Immunizations

Bone marrow-derived dendritic cells (DCs) were generated as previously described 10) by culturing bone marrow cells from female BALB/c mice for 6–8 days in RPMI 1640 (Gibco, USA) supplemented with 10% heat inactivated fetal calf serum (CSL, Australia), 20 mM HEPES buffer (JRH, USA), 4 mM l-glutamine, 100 U/mL penicillin, 100 mg/mL streptomycin sulfate, 100 mM β-mercaptoethanol (all Sigma, Australia), 1,000 U/mL granulocyte and macrophage colony stimulating factor (GM-CSF, Pharmingen, USA), and 10 ng/mL of interleukin-4 (IL-4, Pharmingen, USA). Female mice were used to avoid rejection of the transferred DCs. DCs were CD11c+MHCII+ and Gr1− with the majority (>80%) co-expressing CD11b+ indicating an inflammatory DC phenotype (data not shown). Harvested DCs were pulsed with 5 µg/mL peptide at 37°C for 1 h. For co-presentation DCs were pulsed with one peptide for 1 h, followed by the variant peptide for a further 2 h. No phenotypic changes were observed (using markers CD11c, CD11b, Gr1, MHCII, CD40, CD80, or CD86) following the peptide pulse for any of the peptides used, and no changes in inflammatory (IL6, TNF, and IL1) or suppressor cytokines (IL-10) in the supernatant, or changes in DC viability (by trypan blue). 10^6^ DCs in 100 µL sterile PBS were injected intradermally into the hind footpads (50 µL per footpad) or into the base of the tail. Experiments involving immunization with one peptide or peptide combination on day 0 followed by booster immunization with another peptide on day 14 included control groups that received the same peptide or peptide combination as single immunization on day 0 only and another group that received a single immunization with the booster peptide on day 14 only. For all sequential immunizations, ELISPOT assays were performed between days 14 and 17 following the last immunization.

### *Ex Vivo* Elispot Assays

ELISPOT assays were carried out in 96 well mixed acetate plates (Millipore, Watford, UK) as previously described (8). MAIPS4510 plates, pretreated with 50 μL/well methanol, were used for IFNγ assays; IL-4 and IL-10 secretion was assessed in MAHAS4510 plates. Plates were coated with 5 µg/mL anti-mouse IFNγ mAb (Mab AN18, Mabtech), 10 µg/mL anti-mouse IL-4 mAb (clone BVD4-1D11, Pharmingen), or 10 µg/mL anti-mouse IL-10 mAb (Pharmingen) and blocked with culture media RPMI 1640 (Gibco, USA) supplemented with 1% heat inactivated mouse serum, 20 mM HEPES buffer (JRH, USA), 4 mM l-glutamine, 100 U/mL penicillin, 100 mg/mL streptomycin sulfate, and 100 mM β-mercaptoethanol (all Sigma, Australia). Around 500,000 freshly isolated splenocytes were cultured in the presence of 10^−5^–10^−11^ M peptide in triplicate or quadruplicate wells. Plates were incubated at 37°C, 5% CO_2_ for 16 h for IFNγ assays, 24 h for IL-4, and 48 h for IL-10 assays. In preliminary assays, cognate responses from splenic T cells from animals immunized with KI, AI, or DI alone were eliminated when splenocytes were depleted of CD8 T cells (Dynabeads, UK) prior to the assay, confirming responses to these minimal CD8 T cell peptide epitopes come from CD8 T cell as expected.

### For *In Vitro* Antagonism Assays

Splenocytes were restimulated with a suboptimal concentration of the index peptide KI for 1 h; the altered variant DI was added at different concentrations and incubated for a further 16 h washed, counted, and plated out as per (9).

Plates were incubated with 1 µg/mL biotinylated anti-mouse IFNγ mAb (Mab R4-6A2-Biotin, Mabtech, Sweden), biotinylated anti-mouse IL-4 mAb (clone BVD6-24G2-biotin, Pharmingen, USA), or biotinylated anti-mouse IL-10 mAb (Pharmingen, USA), followed by 1 µg/mL streptavidin–alkaline phosphatase (AP) (Mabtech, Sweden) for IFNγ assay or 0.1 µg/mL extravidin–AP (Sigma, USA) for IL-4 and IL-10 assays and developed using a colorimetric AP Kit (BioRad, Hercules, CA, USA). The number of spot forming units (SFU) per well was scored using the AID ELISPOT reader (Autoimmun Diagnostika GmbH, Germany) with AID ELISpot software version 2.9 (Autoimmun Diagnostika GmbH, Germany).

### Statistical Analysis

All statistical analyses were carried out using GraphPad Prism (version 6.01). ELISPOT data were analyzed using two-way ANOVA followed by Tukey’s *post hoc* test for comparison of all groups or Dunnett’s *post hoc* test for comparison to the index peptide. The significance level used was (α) = 0.05 for all statistical tests.

## Results

### Co-immunization with Variants KI and DI Impairs the Generation of KI-Specific IFNγ-Secreting CD8 T Cells

**Figure 1:**
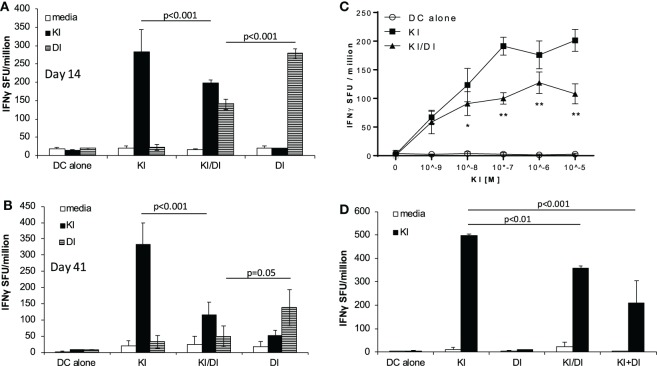
**Impaired IFNγ secretion following co-immunization with peptides KI and DI presented on the same or on different DC**. BALB/c mice were immunized with dendritic cells (DCs) pulsed with either peptide KI or DI alone or with DCs pulsed with KI for 1 h followed by DI for 2 h. Control mice received DCs without peptide. IFNγ secretion was assessed by *ex vivo* ELISpot assay **(A)** 14 days or **(B)** 41 days after immunization. **(C)** IFNγ secretion upon restimulation with peptide KI at concentrations ranging from 10^−9^ to 10^−5^ M was assessed by *ex vivo* ELISpot assay 13 days after immunization. **(D)** BALB/c mice were immunized with DCs pulsed with either peptide KI or DI alone, with DCs pulsed with KI for 1 h then with DI for 2 h (KI/DI), or with a 1:1 mixture of DCs pulsed separately with KI or DI (KI + DI). IFNγ secretion was assessed by *ex vivo* ELISpot 17 days after immunization. Mean spot forming units ± SD are shown (*n* = 3 mice per group). Two-way ANOVA with Tukey’s **(A,B,D)** or Dunnett’s **(C)** multiple comparison test was used to test for statistical significance when comparing all groups or when compared to the group immunized with the index peptide KI, respectively.

To address the question whether co-immunization with epitope variants SYIPSAEKI (KI) and SYIPSAEDI (DI) could broaden the immune response, we immunized BALB/c mice with DCs loaded with both peptides KI and DI. To minimize competition for MHC class I binding clefts, and to ensure KI presentation, DCs were pulsed first with variant KI for 1 h, followed by variant DI for another 2 h at equimolar concentrations (0.5 × 10^−6^ M). Control mice received DCs pulsed with KI or DI alone or without peptide. No KI-specific IFNγ secreting cells were detected in mice immunized with DI-pulsed DCs, confirming that priming with DI did not induce cross-reactive T cells. DCs pulsed with KI alone induced strong KI-specific IFNγ responses, while DCs co-presenting both KI and DI (KI/DI) induced significantly less KI-specific IFNγ-producing T cells (Figure 1A, *p* < 0.001). This significant reduction in KI-specific IFNγ responses was still evident 6 weeks after immunization (Figure 1B, *n* = 3, *p* < 0.001). KI-specific IFNγ responses could not be restored to normal level by using more antigen (Figure 1C, *n* = 3). To rule out peptide competition for H-2K^d^ binding, DCs were also pulsed separately with variant KI or DI for 1 h, washed, and then mixed at 1:1 ratio (KI + DI) immediately before injection. Control mice received (i) DCs pulsed with KI + DI, (ii) DCs pulsed with either variant KI or DI alone as positive controls, or (iii) DCs without peptide as a negative control. Figure 1D shows that KI-specific IFNγ-secreting cells were reduced irrespective of whether DCs had been loaded with KI and DI together (*p* = 0.009) or separately (*p* < 0.001). No KI-specific IFNγ secretion was observed in DI-immunized mice as observed previously. These data suggest that DI interfered with priming of KI-specific IFNγ secreting T cells *in vivo* when presented together with KI on the same or different APC. Therefore, this interference is unlikely to have been caused by peptide competition for MHC binding. Furthermore, no interference with KI-specific T cell priming was observed with DCs co-presenting KI together with the unrelated H-2K^d^-restricted CD8 T-cell epitope NPK^d^ (TYQRTRALV) from the influenza A virus nucleoprotein (aa 147–155) (11), a peptide with strong binding affinity for H-2k^d^ (12, 13). The immune interference observed with KI + DI immunization appeared therefore unlikely to be due to the DC-loading protocol but suggests inhibitory effects were mediated specifically by variant DI.

### Co-immunization with Variants KI and AI Does Not Interfere with KI-Specific or AI-Specific T Cell Priming

**Figure 2:**
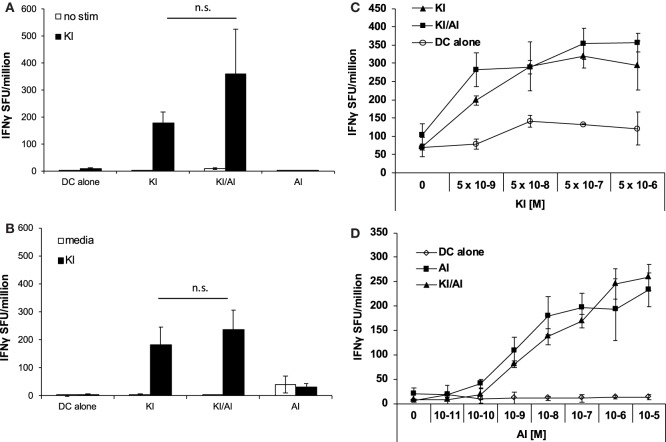
**Co-immunization with KI and AI does not interfere with T cell priming**. BALB/c mice were immunized with dendritic cells (DCs) pulsed with either peptide KI or AI alone or with DC pulsed with KI for 1 h then with AI for 2 h. Control mice received DCs without peptide. **(A)** 12 days or **(B)** 31 days post-immunization IFNγ secretion was assessed by *ex vivo* ELISpot by restimulation with peptide KI at 0.5 µg/mL. **(C)** BALB/c mice were immunized with DCs pulsed with either peptide KI (triangles) or with DC pulsed with KI for 1 h then with AI for 2 h (squares). Control mice received non-pulsed DCs (open circles). IFNγ secretion was assessed by *ex vivo* ELISpot by restimulation with peptide KI at concentrations ranging from 5 × 10^−9^ to 5 × 10^−6^ M 10 days post-immunization. **(D)** BALB/c mice were immunized with DCs pulsed with either peptide AI (triangles) or with DC pulsed with KI for 1 h then with AI for 2 h (squares). Control mice received non-pulsed DCs (open circles). IFNγ secretion was assessed by *ex vivo* ELISpot by restimulation with peptide AI at concentrations ranging from 5 × 10^−9^ to 5 × 10^−6^ M 21 days post-immunization. Mean spot forming units ± SD are shown (*n* = 3 mice per group). Two-way ANOVA with Dunnett’s multiple comparison test was used to test for statistical significance when compared to the groups immunized with the index peptide KI.

When we tested the effect of co-immunization of KI with another variant SYIPSAEAI (AI), we observed a different effect. Using the same immunization strategy as above, DCs were pulsed with KI for 1 h followed by AI for 2 h at equimolar concentration (5 × 10^6^ M) (KI/AI). Control mice were immunized with DCs pulsed with KI and AI alone or without peptide. KI-specific IFNγ responses were assessed by *ex vivo* ELISpot assay. We observed no significant difference in KI-specific IFNγ responses between mice immunized with KI or KI/AI at 2 (Figure 2A) or 5 weeks (Figure 2B) post-immunization. Titration of peptide KI from 5 × 10^−9^ to 5 × 10^−6^ M for *ex vivo* stimulation showed a similar reactivity pattern for KI-immunized mice and KI/AI-immunized mice at all concentrations tested (Figure 2C). Together, these data suggest that epitope variant AI does not interfere with KI-specific T cell priming when presented together with KI *in vivo*, indicating a functional difference between the two altered peptide variants AI and DI. To assess whether KI interfered with AI-specific priming, splenocytes from mice immunized with KI/AI or AI alone were assessed for AI-specific IFNγ responses by *ex vivo* ELISpot assay. Titration of peptide AI showed a similar reactivity pattern for AI- or KI/AI-immunized mice at all concentrations tested (Figure 2D). These results show that co-presentation of peptides KI and AI does not interfere with T cell priming.

**Figure 3:**
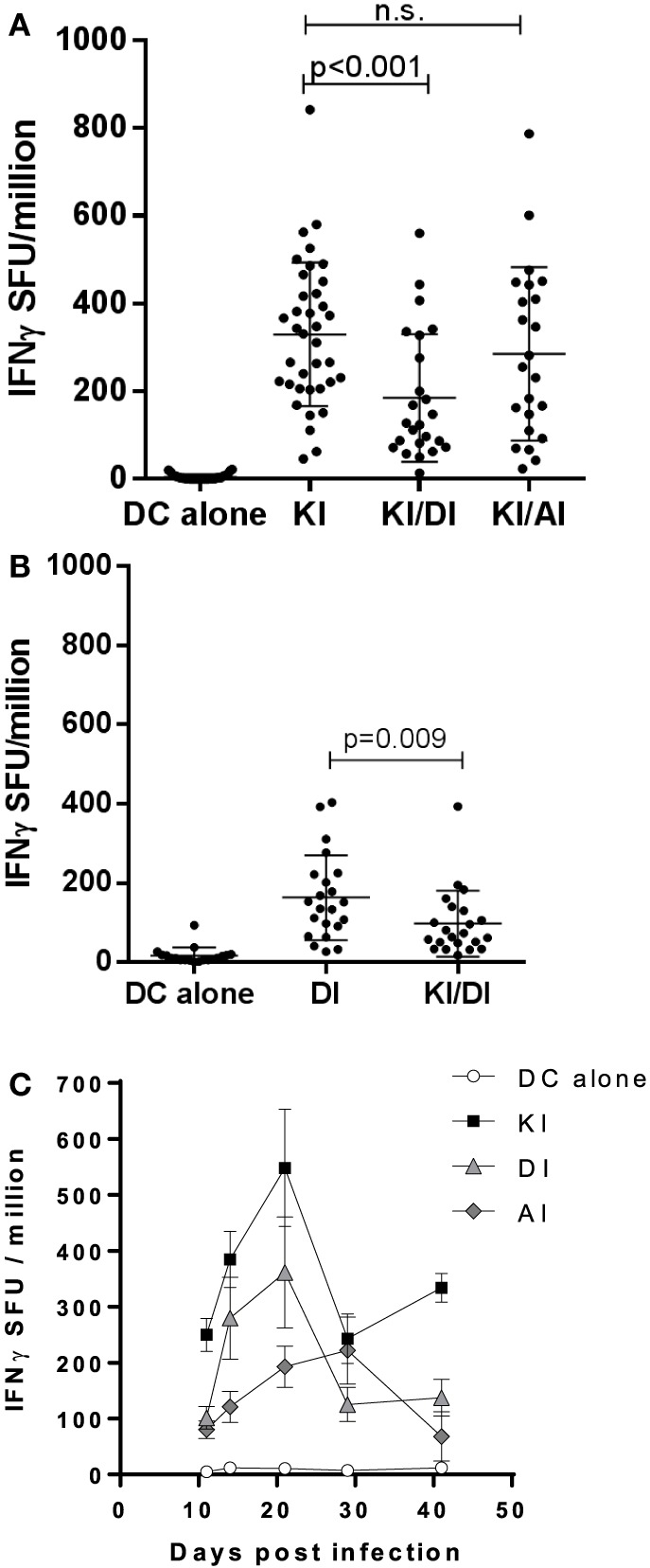
**Pooled data**. **(A)** BALB/c mice immunized with dendritic cells (DCs) pulsed with either peptide KI alone (*n* = 37), with DCs pulsed with KI for 1 h followed by DI for 2 h (*n* = 24) or with DC pulsed with KI for 1 h then with AI for 2 h (*n* = 23). Control mice received DCs without peptide (*n* = 30). KI-specific IFNγ response was assessed by *ex vivo* ELISpot assay. Pooled data from 12 experiments are shown. Dots represent individual mice, and the horizontal line represents the mean ± SD. Data were analyzed using one-way ANOVA followed by Bonferroni’s multiple comparison test. **(B)** BALB/c mice were immunized with DCs pulsed with either peptide DI alone or with DCs pulsed with KI for 1 h followed by DI for 2 h. Control mice received DCs without peptide. DI-specific IFNγ response was assessed by *ex vivo* ELISpot assay. Pooled data from eight experiments are shown. Dots represent individual mice, and the horizontal line represents the mean ± SD. Data were analyzed using one-way ANOVA followed by Bonferroni’s multiple comparison test. **(C)** Mice were immunized with DCs pulsed with KI (black squares), DI (gray triangles), or AI (gray diamonds) alone and IFNγ responses to their respective peptides measured by *ex vivo* ELISpot assay at different time points. KI response is shown for control mice that received DCs without peptide. Mean spot forming units ± SD are shown (*n* = 3–9 mice per time point).

Across experiments, mice immunized with KI-pulsed DC varied in their KI-specific IFNγ responses. Figure 3A shows pooled data from 12 experiments. The mean KI-specific IFNγ response in mice immunized with DC pulsed KI/DI (mean 185 ± 146 SFU/million) was significantly lower compared to those immunized with KI alone (mean 330 ± 164 SFU/million; *p* < 0.001). By contrast, KI-specific IFNγ responses in mice co-immunized with KI/AI (mean 285 ± 197 SFU/million) was comparable to those immunized with KI alone (Figure 3A).

### Variants KI and DI Show Potential Mutual Inhibitory Effects on T Cell Priming

We next tested the effect of KI/DI co-presentation of the priming on DI-specific IFNγ responses after immunization with DCs co-presenting KI and variant DI (KI/DI) or DI alone. Mice immunized with KI/DI had significantly fewer DI-specific IFNγ secreting cells than mice immunized with DI alone (Figure 1A, *p* < 0.001), an effect that was less evident at 6 weeks post-immunization (Figure 1B, *p* = 0.05). Across experiments KI/DI immunization elicited significantly lower DI-specific IFNγ responses (mean 98 ± 83 SFU/million) compared to mice immunized with DC pulsed with DI alone (mean 164 ± 107 SFU/million; Figure 3B; *p* < 0.01). Although we cannot exclude that this effect might have been due to DC being pulsed with KI first followed by DI, this observation suggests that altered peptide variants KI and DI could be mutually antagonistic/immune interfering during T cell priming *in vivo*. KI- or DI-specific IFNγ responses following single immunization with the respective peptide displayed a similar response curve with peak responses elicited 2–3 weeks post-immunization (Figure 3C). It therefore appears unlikely that the reduced IFNγ responses following co-presentation of both peptides should result from different peptide kinetics. The implication for pathogen immune evasion is that a host exposed to both variants simultaneously might generate impaired IFNγ responses to both epitopes. This potential mutual interference was surprising and led us to investigate what would happen if mice were immunized sequentially with peptides KI and DI.

### Variant DI Inhibited KI-Primed Effector T Cells *In Vivo*

**Figure 4:**
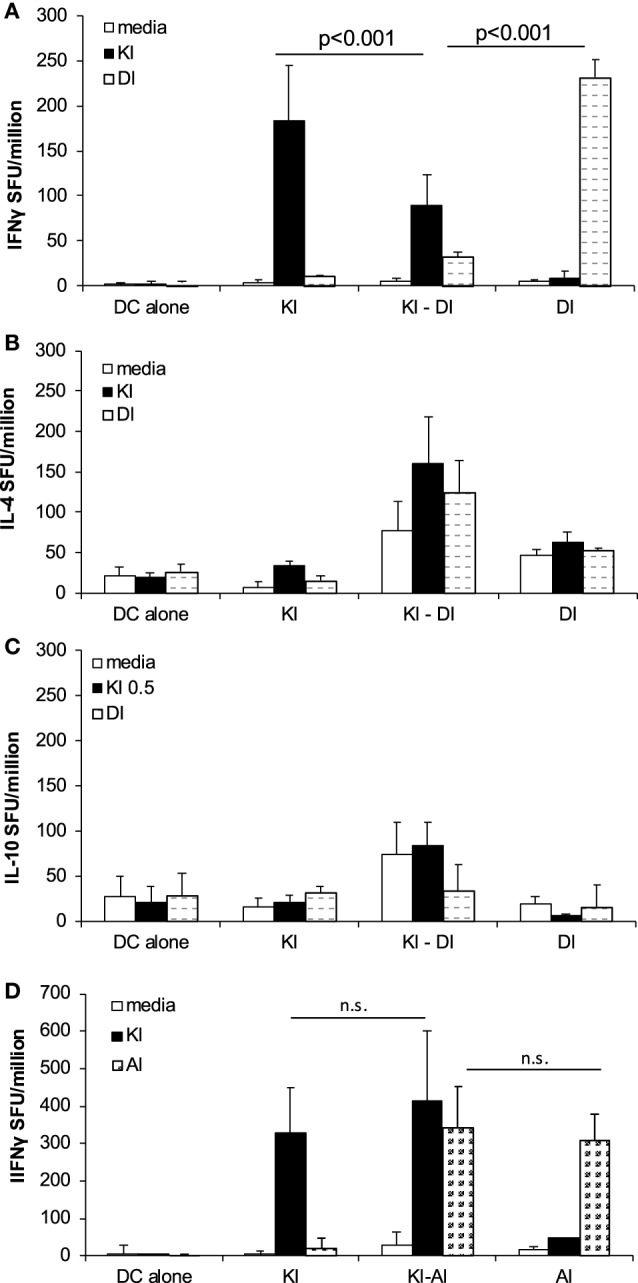
**Immunization with variant DI but not variant AI inhibits KI-specific IFNγ-secreting effector T cells *in vivo***. BALB/c mice were immunized with dendritic cells (DCs) pulsed with KI on day 0 (KI), DCs pulsed with DI on day 14 (DI) or with KI-pulsed DCs on day 0 followed by DI-pulsed DCs on day 14 (KI–DI). The negative control group received two immunizations with non-pulsed DCs 14 days apart. Splenocytes were assessed for KI-specific (black bars) and DI-specific (gray bars) **(A)** IFNγ, **(B)** IL-4, and **(C)** IL-10 responses by *ex vivo* ELISpot assay 17 days after the last immunization (day 31). **(D)** BALB/c mice were immunized with DCs pulsed with KI on day 0 (KI), with DCs pulsed with AI on day 14 (AI) or with KI-pulsed DCs on day 0 followed by AI-pulsed DCs on day 14 (KI–AI). The negative control group received two immunizations with non-pulsed DCs 14 days apart. Splenocytes were assessed for KI-specific (black bars) and AI-specific (gray bars) IFNγ responses by *ex vivo* ELISpot assay 14 days after boost immunization (day 28). Mean spot forming units ± SD (*n* = 3 mice per group). Two-way ANOVA with Tukey’s multiple comparison test was used to test for statistical significance when comparing all groups **(A,B,D)**. Two-way ANOVA with Dunnett’s multiple comparison test was used to test for statistical significance when comparing to the media control (C).

To study *in vivo* effects of DI on KI-primed effector T cells, we primed BALB/c mice with the index peptide KI (d0) and 2 weeks later (d14) injected DCs presenting DI. We called this sequential immunization protocol KI–DI. Control mice received KI-pulsed DCs on day 0 (KI) or DI-pulsed DCs on day 14 (DI) or DCs without peptide (DC alone). KI-specific IFNγ responses were measured 14–17 days after the last immunization by *ex vivo* ELISpot assay. Mice primed with the index variant KI that received no further immunizations showed strong KI-specific IFNγ responses detectable 31 days post-immunization (Figure 4A). Mice receiving the KI–DI sequential immunizations, however, had significantly reduced numbers of KI-specific IFNγ-secreting cells (Figure 4A, black bars; *p* < 0.01). No difference was observed in the numbers of KI-specific IL-4-secreting cells between mice immunized with KI alone or followed by DI 14 days later (Figure 4B, black bars), and no KI-specific IL-10 secretion was detected in any of the groups (Figure 4C). These results suggest that epitope variant DI has an inhibitory effect on polyclonal KI-primed IFNγ secreting but not IL-4 secreting effector T cells *in vivo*.

DI-specific IFNγ responses were also measured after sequential immunization. Interestingly, there were significantly fewer DI-specific IFNγ-secreting cells detected in mice previously primed with KI compared with mice that were not pre-primed (Figure 4A, gray bars, *p* < 0.001). No significant difference in DI-specific IL-4 secretion (Figure 4B) and no DI-specific IL-10 secretion (Figure 4C) were observed in these mice. These results show that priming with the index peptide KI inhibits subsequent priming with the altered peptide variant DI.

No such interference was observed with AI-specific priming, using the above sequential immunization protocol for KI–AI immunization. KI-primed mice showed strong KI-specific IFNγ responses irrespective of whether they were later immunized with AI-pulsed DCs or not (Figure 4D, black bars) with no statistically significant difference in the frequencies of KI-specific IFNγ-secreting effector T cells between KI- and KI–AI-immunized mice. Variant AI had no inhibitory effect on KI-specific IFNγ effector responses *in vivo*. Similarly, KI-primed mice raised competent AI-specific IFNγ-secreting cells in response KI–AI immunization, similar to AI-immunized mice (Figure 4D, gray bars).

### KI-Specific IFNγ Responses Were Inhibited in the Presence of Epitope Variant DI *In Vitro*

**Figure 5:**
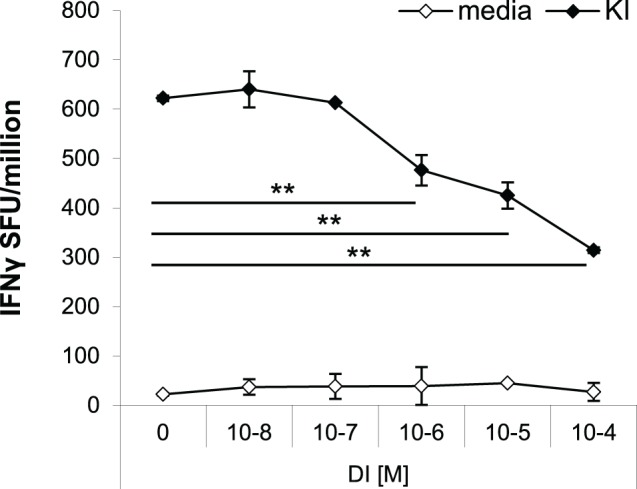
**Variant DI antagonizes KI-specific IFNγ secretion *in vitro***. BALB/c mice were immunized with 10^6^ dendritic cells (DCs) pulsed with peptide variant KI or with DCs without peptide as negative controls. IFNγ responses were measured 21 days post-immunization by *ex vivo* ELISpot assay. Splenocytes were restimulated with the index peptide KI at 10^−8^ M for 1 h, followed by the altered peptide variant DI at different concentrations (10^−8^–10^−4^ M). Mean spot forming units ± SD are shown (*n* = 3 mice per group). Two-way ANOVA with Dunnett’s multiple comparison test was used to test for statistical significance when compared to the groups immunized with the index peptide KI.

The effects of antagonistic peptides have been studied on both clonal and polyclonal effector T cell responses (9, 14). Peptide variant DI has previously been shown to partially antagonize KI-specific IFNγ secretion *in vitro* in splenocytes from mice immunized with KI-expressing protein particles derived from a yeast retrotransposon (TyS3) (9). Here, we confirm that peptide DI inhibits KI-specific effector T cell responses *in vitro* using a protocol optimized in previous studies to control for the issue of competition of peptides for binding to MHC (9, 15). For the antagonism assay, splenocytes from KI-immunized mice were incubated with the suboptimal KI concentration of 10^−8^ M (0.01 µg/mL), and after 1 h DI was added at concentrations ranging from 10^−8^ to 10^−4^ M. Figure 5 shows that addition of the altered variant DI significantly inhibited KI-specific IFNγ secretion from concentrations as low as 10^−6^ M (0.1 µg/mL). The inhibitory effect increased with increasing DI concentration, but inhibition never exceeded 60% of the total IFNγ response to the index peptide KI.

### KI/AI-Primed Cells Were Not Susceptible to *In Vivo* Immune Interference by DI

**Figure 6:**
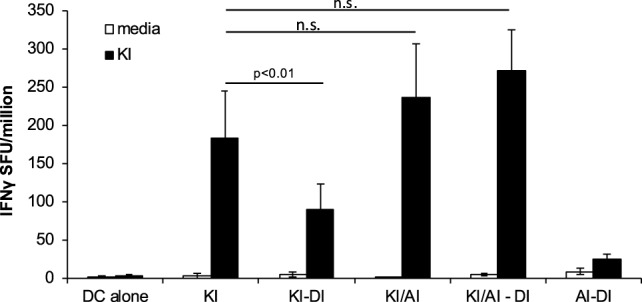
**KI/AI-primed cells are resistant to DI-induced immune interference**. BALB/c mice were immunized on day 0 with dendritic cells (DCs) pulsed with either KI or AI or with DCs pulsed with KI for 1 h followed by AI for another 2 h. On day 14, three groups received sequential immunization with DI-pulsed DCs (KI–DI, KI/AI–DI, and AI–DI). The negative control group received two immunizations with non-pulsed DCs 14 days apart. Splenocytes were assessed for KI-specific IFNγ responses by *ex vivo* ELISpot assay 17 days after boost immunization (day 31). Mean spot forming units ± SD are shown (*n* = 3 mice per group). Two-way ANOVA with Dunnett’s multiple comparison test was used to test for statistical significance when compared to the groups immunized with the index peptide KI.

Similar frequencies of KI-specific IFNγ-secreting cells were detected in the spleens from KI-immunized and KI/AI-immunized mice, although mice primed with KI or AI individually showed no cross-reactivity to the other variant (data not shown). In order to determine whether KI-specific CD8 T cells, induced by co-immunization with KI/AI, would have a similar or distinct pattern of cross-reactivity and/or susceptibility to immune interference to those induced with KI alone, mice were immunized with either KI/AI together, or KI alone on DCs (day 0), and 2 weeks later with DI-pulsed DCs (KI/AI–DI and KI–DI, respectively). Control mice received DCs pulsed with KI or without peptide. KI-specific IFNγ responses were measured 2–3 weeks later by *ex vivo* ELISpot assay. Three experiments were performed to assess the effect of variant DI on KI-specific IFNγ responses from KI/AI-primed splenocytes. The responses of mice immunized with KI were as expected susceptible to immune interference by subsequent immunization with DI (Figure 6). By contrast, we detected no significant difference in KI-specific IFNγ responses between KI/AI-primed mice that received a subsequent immunization with DI-pulsed DCs on day 14 and those that did not (Figure 6). Mice immunized with DCs presenting the variant AI alone on day 0 followed by immunization with DCs pulsed with variant DI on day 14 had no detectable KI-specific IFNγ responses (Figure 6), consistent with the previously shown lack of cross-reactivity. Our results show that KI/AI immunization, in contrast to immunization with KI or AI alone, induced T cells that recognize KI and AI, while no longer being susceptible to being turned off by immunization with the antagonistic DI APL, rendering the response therefore resistant to this type of immune interference.

## Discussion

The present study shows that immunization with combinations of highly related immunogenic variant peptides (KI, DI, and AI), such as those found naturally in pathogen populations with highly polymorphic T-cell epitopes, can have highly variable, and unpredictable, outcomes, with some combinations capable of decreasing immunity to both immunizing peptides (KI and DI). Moreover, immunizing sequentially with such peptides was shown to be capable of turning off responses, again to both immunizing T-cell epitope variants (KI and DI). The existence of “sequential immune interference” has theoretical consequences for pathogen population structures. It may be that polymorphic variants provide a survival advantage to the pathogen population that allows the species to evade the host immune response. By contrast, co-immunization of KI and AI had apparently little effect on the initial induction of immunity to either epitope, but, unexpectedly, the KI-specific responses generated were no longer susceptible to being turned off by subsequent immunization with DI. This finding offers a new practical strategy to elicit robust immune responses against polymorphic variants (by co-immunizing with an index epitope and a carefully selected “potentiator” epitope), which are then further resistant to being turned off by natural polymorphic variants capable of immune interference. *In vivo* challenge experiments with *P. berghei* sporozoites are needed to quantify the extent to which protective immunity is altered in this model.

Each of the three CD8 T-cell epitopes selected for study (KI, DI, and AI) induced high immune responses to themselves, but not to the other two epitopes, thus at face value they represent simple non-cross-reactive T-cell epitopes. However, co-immunization with DC pre-pulsed with KI and DI together, significantly reduced responses to both epitopes, with this mutual immune interference observed even when KI and DI were injected separately on separately peptide pulsed DC. There was no correlation between the magnitude of induced KI response and the level of interference by DI. Furthermore, the DI peptide was also shown to be capable of turning off effector KI-specific responses in a manner consistent with a classical APL antagonist. However, the fact that immune interference was observed even when presenting the epitopes on separate DC shows that the immune interference observed herein is not acting *via* classical mechanisms used by APL antagonism, such as alterations of the TCR:MHC immune-synaptosome structure (16). Further studies will be required to fully explore the extent to which immune interference by KI and DI is potentially fully reciprocal under a range of co-presentation and sequential immunization combinations.

The activity of some APL antagonist peptides has been shown to depend on a high off-rate in relation to MHC binding, but such mechanisms are unlikely to underlie the observed immune interference described in this study, since we have previously shown similarly high affinity binding to MHC for KI, DI, and AI (1). Temporal separation of stimulation by KI and DI by sequential immunization also resulted in reduced immunity demonstrating that immune interference by DI acts by stimulating KI-specific T cells to change their activity profile. Such switching of an activity profile has been observed *in vitro* for CD4 T-cell epitopes from *P. falciparum*. In this case, stimulation with a variant polymorphic epitope of an index peptide converted IFNγ-producing cells into IL-10 producers; a property demonstrated with human T cell clones to Th2R region variants of CS (2). In our study, immunization with DI did not appear to switch KI-specific responses from IFNγ to IL-10, or convert them into IL-4-producing Th2 cells, which have been noted as previous APL mechanisms capable of down modulating Th1 and cytotoxic immunity. These results do not exclude the hypothesis that stimulation with DI promotes other, less obvious, immunosuppressive cell populations, as well as potentially T cell apoptosis, anergy, or partial activation, related in a number of other studies with low TCR affinity interactions or disturbed TCR stimulating signaling micro-domains [reviewed in Ref. (17, 18)].

By using a combination of the index variant KI together with the “potentiator” peptide AI, we have demonstrated the induction of an immune response that is resistant to immune interference by DI stimulation. This finding lays the experimental foundations for a practical immunization strategy that could induce robust and long-lasting immune responses in the malaria setting. Thus, index epitopes could be paired with potentiator epitopes in a single immunization to induce a robust immune response to a polymorphic epitope, which would be predicted to persist even in vaccinees challenged by repeated pathogen stimulation. This is potentially of great significance for those living in malaria-endemic regions and exposed to persistent reinfection by polymorphic parasites capable of turning off immunity using immune interference (1). Moreover, combinations of engineered and natural variants may offer a new path for vaccines against many different pathogens, to create new modalities of cross-reactive responses.

## Author Contributions

GM and MP designed the study, interpreted the data, and drafted the manuscript. GM performed experiments and analyzed data. KF and RS critically revised the manuscript.

## Conflict of Interest Statement

The authors declare that the research was conducted in the absence of any commercial or financial relationships that could be construed as a potential conflict of interest.
